# Post-COVID-19 condition: systemic inflammation and low functional exercise capacity

**DOI:** 10.3389/fnut.2024.1295026

**Published:** 2024-03-14

**Authors:** Gabriela Salim de Castro, Leonardo R. Gama, Alexandre Ferreira Ramos, Guilherme Gatti da Silva, Alexandre Abilio de Souza Teixeira, Edecio Cunha-Neto, Heraldo Possolo de Souza, Suely K. Marie, Leda L. Talib, Verônica Coelho, Jorge Kalil, Adriana Ladeira de Araujo, Ana Paula Ritto, Alessandro Rodrigo Belon, Amanda Soares Santos, Ana Paula Noronha Barrére, Márcio V. Y. Sawamura, Celina Almeida Lamas, Bruno Guedes Baldi, Carlos R. R. Carvalho, Leslie Domenici Kulikowski, Rodolfo Furlan Damiano, Marta Imamura, José Cesar Rosa Neto, Fabio S. Lira, José Pinhata Otoch, Euripedes Constantino Miguel, Linamara Battistella, Orestes V. Forlenza, Geraldo Busatto, Marilia Seelaender

**Affiliations:** ^1^Cancer Metabolism Research Group, Departamento de Cirurgia and LIM 26-HC da Faculdade de Medicina da Universidade de São Paulo, São Paulo, Brazil; ^2^Centro de Pesquisa Translacional em Oncologia, Instituto do Câncer do Estado de São Paulo, Universidade de São Paulo, São Paulo, Brazil; ^3^Escola de Artes, Ciências e Humanidades, Universidade de São Paulo, São Paulo, Brazil; ^4^Instituto de Ciencias Biomedicas, Departamento de Biologia Celular e do Desenvolvimento, São Paulo, Brazil; ^5^Departamento de Clínica Médica, Universidade de São Paulo FMUSP, São Paulo, Brazil; ^6^Instituto de Investigação em Imunologia, Instituto Nacional de Ciência e Tecnologia (III-INCT), São Paulo, Brazil; ^7^Departamento de Emergências Médicas, Universidade de São Paulo FMUSP, São Paulo, Brazil; ^8^Departamento de Neurologia, Universidade de São Paulo FMUSP, São Paulo, Brazil; ^9^Laboratorio de Citogenomica, Departamento de Patologia, Faculdade de Medicina da Universidade de São Paulo (FMUSP), São Paulo, Brazil; ^10^Laboratório de Imunologia, Instituto do Coração, Hospital das Clinicas HCFMUSP, Faculdade de Medicina, Universidade de São Paulo, São Paulo, Brazil; ^11^Diretoria Executiva dos LIMs, Faculdade de Medicina da Universidade de São Paulo, São Paulo, Brazil; ^12^Centro de Inovação InovaHC do Hospital das Clínicas HCFMUSP, Faculdade de Medicina, Universidade de São Paulo, São Paulo, Brazil; ^13^Instituto de Radiologia (InRad), Hospital das Clínicas, Faculdade de Medicina, Universidade de São Paulo (HCFMUSP), São Paulo, Brazil; ^14^Divisão de Pneumologia do Instituto do Coração (InCor), Hospital das Clínicas, Faculdade de Medicina, Universidade de São Paulo (HCFMUSP), São Paulo, Brazil; ^15^Departamento e Instituto de Psiquiatria, Hospital das Clínicas da Faculdade de Medicina da Universidade de São Paulo HCFMUSP, São Paulo, Brazil; ^16^Departamento de Medicina Legal, Bioetica, Medicina do Trabalho e Medicina Fisica e Reabilitacao, Faculdade de Medicina, Universidade de São Paulo, São Paulo, Brazil; ^17^Exercise and Immunometabolism Research Group, Programa de Pós-Graduação em Ciências do Movimento, Departamento de Educação Física, Faculdade de Ciências e Tecnologia, Universidade Estadual Paulista (UNESP), Presidente Prudente, São Paulo, Brazil; ^18^Universidade de São Paulo Hospital Universitario, São Paulo, Brazil; ^19^Instituto de Medicina Fisica e Reabilitacao, Hospital das Clinicas HCFMUSP, Faculdade de Medicina, Universidade de São Paulo, São Paulo, Brazil

**Keywords:** COVID-19, post-COVID-19 condition, post-acute sequelae of SARS-CoV-2 infection, PASC, long COVID, inflammation, cytokines, fatigue

## Abstract

**Introduction:**

Post-COVID-19 condition (PCC) is characterised by a plethora of symptoms, with fatigue appearing as the most frequently reported. The alterations that drive both the persistent and post-acute disease newly acquired symptoms are not yet fully described. Given the lack of robust knowledge regarding the mechanisms of PCC we have examined the impact of inflammation in PCC, by evaluating serum cytokine profile and its potential involvement in inducing the different symptoms reported.

**Methods:**

In this cross-sectional study, we recruited 227 participants who were hospitalised with acute COVID-19 in 2020 and came back for a follow-up assessment 6–12 months after hospital discharge. The participants were enrolled in two symptomatic groups: Self-Reported Symptoms group (SR, *n* = 96), who did not present major organ lesions, yet reported several debilitating symptoms such as fatigue, muscle weakness, and persistent loss of sense of smell and taste; and the Self-Reported Symptoms and decreased Pulmonary Function group (SRPF, *n* = 54), composed by individuals with the same symptoms described by SR, plus diagnosed pulmonary lesions. A Control group (*n* = 77), with participants with minor complaints following acute COVID-19, was also included in the study. Serum cytokine levels, symptom questionnaires, physical performance tests and general clinical data were obtained in the follow-up assessment.

**Results:**

SRPF presented lower IL-4 concentration compared with Control (*q =* 0.0018) and with SR (*q =* 0.030), and lower IFN-α2 serum content compared with Control (*q* = 0.007). In addition, SRPF presented higher MIP-1β serum concentration compared with SR (*q =* 0.029). SR presented lower CCL11 (*q =* 0.012 and *q =* 0.001, respectively) and MCP-1 levels (*q =* 0.052 for both) compared with Control and SRPF. SRPF presented lower G-CSF compared to Control (*q* = 0.014). Female participants in SR showed lower handgrip strength in relation to SRPF (*q =* 0.0082). Male participants in SR and SRPF needed more time to complete the timed up-and-go test, as compared with men in the Control group (*q =* 0.0302 and *q =* 0.0078, respectively). Our results indicate that different PCC symptom profiles are accompanied by distinct inflammatory markers in the circulation. Of particular concern are the lower muscle function findings, with likely long-lasting consequences for health and quality of life, found for both PCC phenotypes.

## 1 Introduction

Post-COVID-19 condition (PCC) is characterised by alterations that cannot be explained by other diagnoses, which last for a minimum of 2 months and occur typically 3 months after SARS-CoV-2 infection (1). Bearing in mind that the affected population is, in average, 40 years old (2), the extended impact of the pandemic is presumed to be long-lasting, with repercussions for public health and global economy. The reported symptoms vary from fatigue and pain to organ lesion/loss of function, and many patients describe a plethora of neurological alterations that markedly compromise quality of life (3).

In the severe form of the acute disease, exacerbated circulation of inflammatory factors, addressed frequently as “cytokine storm” (4, 5) was a common finding in the period prior to the beginning of vaccination. Unfortunately, however, vaccination seems to confer only partial protection against PCC (6). Given the relevance of inflammatory mediation in acute COVID-19, it is not bold to anticipate that PCC might be also associated with specific systemic and local inflammatory changes, in particular when it is clear that pain and fatigue are the most frequently reported symptoms. Furthermore, the combination of inflammation (both acute and chronic forms) and hospitalisation impacts body composition, inducing loss of muscle function in several scenarios (7, 8). As COVID-19 patients face the same challenges, it is of concern that lean mass loss may very likely be present in PCC, potentially affecting function, quality of life and the response to disease in the future. Furthermore, low muscle mass and function have been associated with poorer outcomes in patients with acute COVID-19 (9, 10). It has also been reported that hospitalisation-associated muscle loss in patients with COVID-19 correlates with fatigue and low muscle mass 6 months after hospital discharge (11). Hanson et al. (12), in a recent meta-analysis, report fatigue to be the main complaint of 51% of the individuals with PCC.

The potential aftermath of COVID-19 may reach a considerable segment of the population, as one in eight patients who had the disease are affected by persistent symptoms (13). It is therefore mandatory that we learn how to detect and manage long-lasting post-acute disease alterations and propose tools for efficient diagnosis, as well as specific treatment strategies. An evaluation of the plasma metabolome of patients with PCC showed alterations in metabolites related to mitochondrial dysfunction, 2 years after SARS-CoV-2 infection, indicating metabolic alterations persistence (14). Furthermore, cognitive symptoms are highly prevalent in people with PCC, but in a previous study with the same cohort, we failed to find clear association between systemic inflammation and the specific neuro-cognitive alterations (15).

The group has previously demonstrated the co-occurrence of several symptoms in volunteers with PCC with possible common underlying traits. It was found that fatigue, as well as psychiatric and cognitive manifestations were the most discriminative symptoms, indicating that these alterations tend to occur together in a cohort of 749 patients who were hospitalised due to COVID-19 (16). This latent trait—co-occurrence of fatigue, psychiatric and cognitive manifestations—as identified through factor analysis, was also associated with body weight loss, poor physical performance, and persistent inflammation, with high C-reactive protein (CRP) and D-dimer (16) content in the circulation. However, no association was found between these inflammatory markers and any individual symptoms of PCC (16). The presence of long-lasting symptoms was associated with physical inactivity after hospital discharge (17). The occurrence of one or more PCC symptoms indicated greater odds for physical inactivity compared to volunteers without any persistent symptoms (17).

In this study, we aimed at demonstrating distinct inflammatory profiles of PCC presenting different symptom-based phenotypes. This study has explored serum cytokine profile, gender differences and clinical and functional assessment, in a cohort of 227 participants. We herein report inflammatory changes and muscle function alterations associated with two distinct PCC phenotypes, according to the patients’ clusters of symptoms (self-reported symptoms and self-reported symptoms in concomitance with decreased pulmonary function); the results were compared with those of control participants, who were hospitalised with acute COVID-19, but with minor complaints after hospital discharge. These analyses are of relevance as they shed light and explore the mechanisms and variations of PCC manifestations. Furthermore, we sought to identify biomarkers associated with self-reported symptoms and functional assessments in men and women separately, providing means for precise medicine diagnosis and treatment.

## 2 Methods

### 2.1 Ethics approval and consent to participate

This study integrates the results of several research projects within Hospital das Clínicas, Faculdade de Medicina, Universidade de São Paulo (HCFMUSP). All projects were approved by the HCFMUSP Ethics Committee (approval numbers: 4.270.242, 4.502.334, 4.524.031, 4.302.745 and 4.391.560). One of the studies is registered at the Registro Brasileiro de Ensaios Clínicos – REBEC.^1^ Informed consent was obtained from all participants in the follow-up assessment.

### 2.2 Study design

We conducted a cross-sectional study with adult participants who went through a follow-up assessment 6–12 months after hospitalisation due to acute COVID-19 (first wave in 2020). The participants were then assigned to distinct phenotypic groups, according to clinical criteria. Clinical data from the hospitalisation period were also employed to describe the groups.

### 2.3 Study population

We opted for broad inclusion criteria, hoping for as many survivors of the first wave of COVID-19 as possible. Thus, the inclusion criteria were: survival 6 months after hospitalisation, hospital stay of at least 24 h, age > 18 years, and confirmed COVID-19 (described in the Supplementary material). Patients with hospital stays shorter than 24 h were not enrolled owing to incomplete databases. Exclusion criteria were: nosocomial COVID-19 infection; previous diagnosis of dementia or end-stage cancer; individuals living in long-term care facilities or with poor mobility, uncapable to leave home after 6 months from hospital discharge; suspected reinfection at the time of follow-up. Patients were invited to participate by telephone and then scheduled for in-person visits.

**Figure 1:**
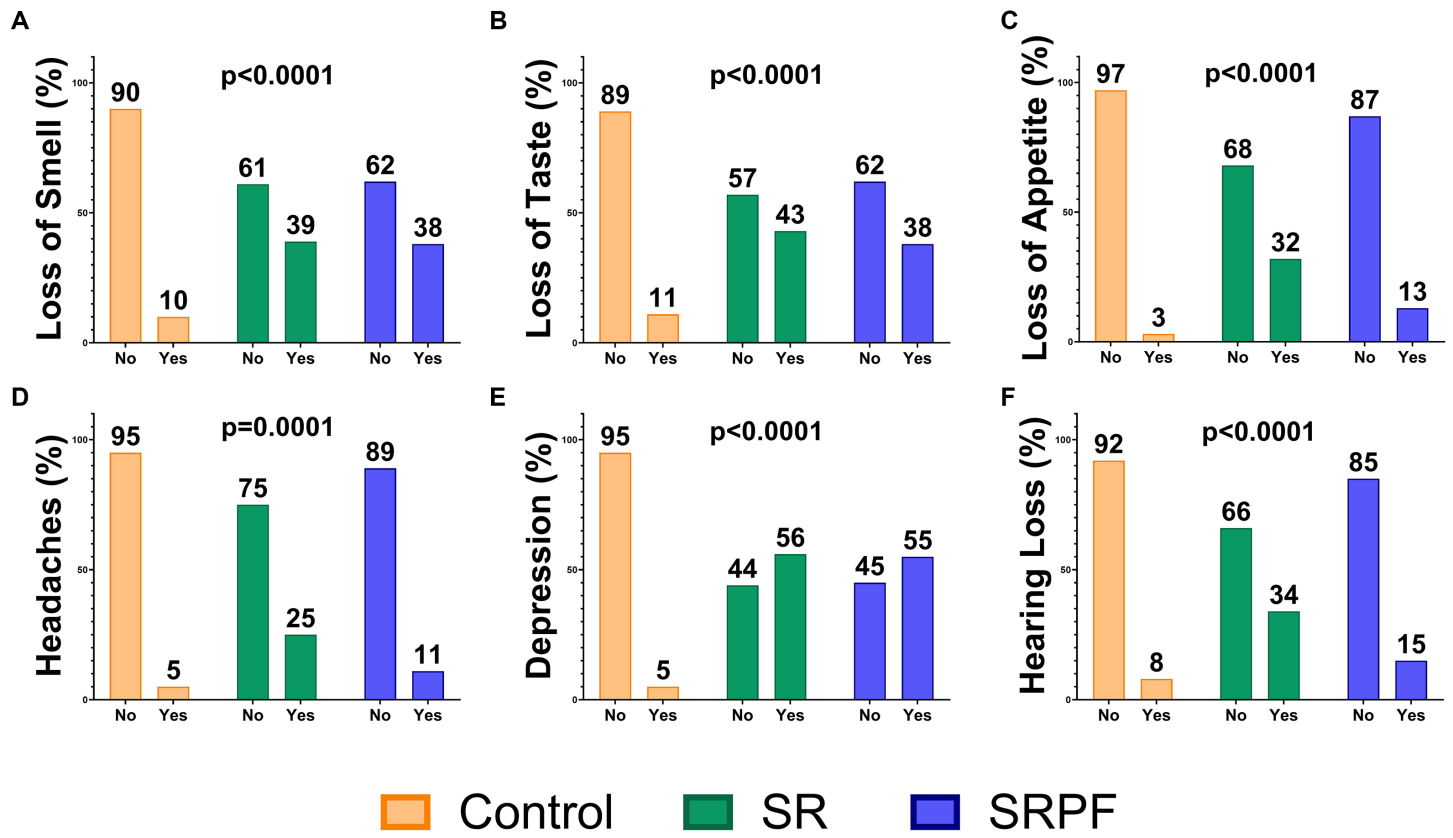
Signs and symptoms in the follow-up assessment. Data are presented as the percentage of patients in the group. **(A)** Loss of Smell; **(B)** Loss of Taste; **(C)** Loss of Appetite; **(D)** Headache; **(E)** Depression; **(F)** Hearing Loss. Comparisons were performed using the Chi-squared test. SR, Self-Reported Symptoms group; SRPF, Self-Reported Symptoms and decreased Pulmonary Function group.

The groups were defined based upon signs and symptoms shown at the follow-up assessment. The participants included in the Control group (total of 77: 29 females and 48 males) should not present decreased lung function and/or lung lesions, should not report fatigue, should not present three or more of the following symptoms: loss of memory, anxiety, lack of concentration, post-traumatic stress, insomnia, and depression. Figure 1 presents the frequency of some of the symptoms evaluated in the follow-up assessment. The Self-Reported Symptoms group (SR, total 96: 66 women and 30 men) was composed by individuals who presented, at the follow-up assessment, a plethora of self-reported alterations (detailed in Figure 1 and Supplementary Figure 3) alongside fatigue; all these symptoms were not noticeable/present previous to COVID-19 diagnosis. The Self-Reported Symptoms and decreased Pulmonary Function group (SRPF, total of 54: 31 women and 23 men) was comprised of volunteers who were diagnosed with decreased lung function, together with self-reported alterations at the follow-up assessment, which should include fatigue and/or dyspnoea. Pulmonary lesions were diagnosed by frontal and lateral chest X-ray and by the spirometry test, and pulmonary capacity was considered deficient when forced vital capacity was lower than 80% of the expected (16).

### 2.4 Clinical evaluation

Data from the hospitalisation period were obtained from the medical records of each volunteer. A detailed description of the recruitment and follow-up assessment was previously given by Busatto et al. (18). Post-COVID-19 self-declared symptoms assessment was provided by answers to direct questions (yes/no answer) or standardised scales – the questions appear as supplemental material in Busatto et al. (16). In addition, patients were submitted to anthropometric measurements (body weight, height, body mass index [BMI]). All participants received orientation about the dyspnoea Borg-scale test, in which participants had to perform a 1-min sit-to-stand test. Oxygen saturation was measured before and after the sit-to-stand test. Participants also performed the timed up-and-go test and handgrip strength measurement (20).

Blood was collected after 12 h fast, while plasma and serum were obtained following blood centrifugation and kept in a -80°C freezer. Blood cell count was assessed with an automated method (21), and glycated haemoglobin was quantified in venous blood (22). CRP (21), D-dimer (21), and ferritin (23) were quantified in the serum following standardised procedures.

### 2.5 Multiplex analysis

The circulating cytokine profile of 227 patients was examined with the Multiplex system employing serum samples obtained in the follow-up assessment. The analysis was carried out with the Human Cytokine/Chemokine Magnetic Bead Panel Cat. HCYTMAG-60 K-PX30 (Merck-Millipore) to evaluate: granulocyte-colony-stimulating factor (G-CSF), granulocyte-macrophage colony-stimulating factor (GM-CSF), interferon (IFN) -α2, IFN-γ, interleukin (IL) -1α, IL-1β, IL-1ra, IL-2, IL-3, IL-4, IL-5, IL-6, IL-7, IL-8, IL-10, IL-12p40, IL-12p70, IL-13, IL-15, IL-17A, interferon gamma-induced protein 10 (IP-10), monocyte chemoattractant protein (MCP) -1, macrophage inflammatory protein (MIP) -1α, MIP-1β, tumour necrosis factor (TNF) -α, TNF-β, vascular endothelial growth factor (VEGF), C-C motif chemokine 11 (CCL11).

Brain-derived neurotrophic factor (BDNF) was assessed employing enzyme-linked immunosorbent assay (ELISA; Catalogue nº. Dy248, R&D System, Minneapolis, MN, United States). The results are expressed as ng/mL.

### 2.6 Statistical analysis

All tests were two-tailed with a significance level of 0.05 and performed using GraphPad Prism version 8.0 for Windows (GraphPad Software, San Diego, CA, United States) and GNU R (version 4.3) (24). Normality and homoscedasticity were evaluated using the D’Agostino & Pearson omnibus normality test, Shapiro–Wilk normality test and Kolmogorov–Smirnov normality test. Numeric variables are expressed as median (interquartile range) and were assessed using the Kruskal-Wallis test followed by Dunn’s test, when data were not normally distributed. Parametric data were analysed using one-way ANOVA with Tukey *post-hoc* test and are expressed as mean and standard deviation. Cytokine concentrations required transformation to minimise batch effect and were analysed as log-transformed levels using Kruskal-Wallis’s test with Dunn’s *post-hoc* test. Categorical parameters were evaluated using the Chi-squared test.

Considering that the follow-up interval for patients’ assessment was different among groups (Supplementary Figure 2), we decided to perform the statistical analysis including this interval as a correction factor. A detailed description of the statistical analysis is in the Supplementary material.

#### 2.6.1 Numerical factors

BDNF levels, cytokine levels (number of variables = 17), laboratory tests (number of variables = 8), vital signs (number of variables = 7) and functional performance tests (number of variables = 5) were compared among groups in a single battery of tests.

##### 2.6.1.1 Distribution and central tendency comparisons between groups

Kruskal-Wallis test by ranks was used to assess differences in the distribution of each variable among groups. To deal with multiple comparisons, we performed *p* value adjustment and calculated the False Discovery Rate (FDR). Both values are reported in the results’ tables. The expected number (E) of false discoveries (V) was set to less than 1.0 by choosing a fitting FDR threshold (t) for each battery of test. Results with an FDR equal to or less than the threshold of t = 0.087, which translates to an expected number of false discoveries of E[V(t)] = 0.87 in this case, were considered positive. For these, we compared central tendencies (medians) between pairs of groups with Dunn’s *post-hoc* test. In this step, rejection of the null hypothesis for test results with an FDR equal to or less than t = 0.052, which gives E[V(t)] = 0.88, was adopted as criterion.

##### 2.6.1.2 Second analysis, stratified by sex and adjusted for age

Sex and age are crucial factors when evaluating physical performance, therefore, we also conducted a sex-stratified analysis, with an additional adjustment by age for the functional tests and glycated haemoglobin concentration.

We stratified the dataset by sex, obtaining a subset of female participants divided in groups of sizes n(SR) = 69, n(SRPF) = 31 and n(Control) = 29, and a subset of male participants in groups of sizes n(SR) = 28, n(SRPF) = 23, n(Control) = 48. After stratification, glycated haemoglobin, vital signs, and functional performance tests were adjusted for age, adopting a linear model.

Kruskal-Wallis test results were considered positive if showing an FDR equal to or less than *t* = 0.076, with E[V(t)] = 0.60 for this battery. We applied Dunn’s test to these variables and rejected the null hypothesis for results with an FDR equal to or less than *t* = 0.063, which gives E[V(t)] = 0.89.

##### 2.6.1.3 Multiple linear regressions

We further investigated whether the cytokines levels could explain the variation in the number of self-reported symptoms or the performance in the physical functional tests. Cytokines included in the model were not linearly correlated. The symptoms “chest pain” and “taste loss” were excluded from the counting due to the high proportion of missing observations. Normality of residuals and homoscedasticity were both present in the model.

## 3 Results

### 3.1 Baseline parameters of the study population at hospital admission during acute COVID-19

The SR group was younger than SRPF (*p* = 0.0226), as shown in Table 1. Furthermore, SR presented female predominance (69%), as compared with the Control group, with, in turn, more men (62%) than women. No differences among groups were observed regarding body weight and BMI, at hospital admission. Serum CRP, D-dimer, and glucose were higher in SRPF, compared with the two other groups (CRP – *p* = 0.0050 vs. Control and *p* < 0.0001 vs. SR; D-dimer – *p* = 0.0003 vs. Control and *p* = 0.0004 vs. SR; glucose – *p* < 0.0001 for both). SRPF also presented higher serum urea and creatinine compared with SR (*p* = 0.0091 and *p* = 0.0058, respectively). SR, on its turn, showed lower circulating creatinine compared with Control (*p* = 0.0158). SR and SRPF showed lower haemoglobin content (*p* = 0.0011 and *p* = 0.0002, respectively) and haematocrit (*p* = 0.0120 and *p* = 0.0133, respectively), compared with the Control group. SRPF presented a higher neutrophil count compared with SR (*p* = 0.0206). No differences were observed for leucocyte, lymphocyte, and platelet counts. SRPF was composed by a higher number of patients with hypertension (67%), obesity (54%) and diabetes (57%), and the number of patients in this group that required intubation during acute disease (78%) was equally higher compared with the other groups. In agreement, SRPF patients had longer hospital stays (*p* < 0.0001 for both) and more days requiring intubation (*p* < 0.0001 for both), compared with the other groups.

**Table 1:** General parameters at the beginning of hospitalisation due to COVID-19.

	Control	SR	SRPF	Value of *p* (respectively)
Age	52.96 ± 15.20	48.33 ± 13.37	54.64 ± 12.30†	0.0226^A^
Sex (female/male)	29/48	66/30	31/23	0.0002^C^
Body weight at admission (kg)	85.5 [75; 95]	75.5 [66; 91.85]	80 [67; 95]	0.1319^K^
BMI at admission (kg/m^2^)	29.9 [26.25; 36.75]	29.45 [25.38; 37.00]	30.3 [24.9; 37.9]	0.8405^K^
CRP (mg/dL)	110.3 [56.8; 208.9]	81.45 [39.85; 180.8]	204.2 [81.6; 298.3]*^,^†	0.0050; <0.0001^K^
D-dimer (mg/L)	797 [516; 1,300]	741 [384.5; 1778]	1,568 [870.5; 3,565]*^,^†	0.0003; 0.0004^K^
B-type natriuretic peptide (pg/mL)^/^	306 [135; 881]	213 [36.25; 2,829]	383 [144.5; 1,060]	0.5416^K^
Glucose^//^	123 [105; 159]	130 [100; 183]	183 [133; 271.5]*^,^†	<0.0001; <0.0001^K^
AST (U/L) ^///^	43.5 [28.75; 64.25]	36 [23; 49]	44.5 [32; 59.5]	0.0362^K^
ALT (U/L) ^///^	43 [22.75; 65.25]	31 [21; 51]	36.5 [26; 47.5]	0.1416^K^
Urea (mg/dL)	36.5 [24; 56]	28 [20; 43.5]	42 [26; 72]†	0.0091^K^
Creatinine (mg/dL)	0.96 [0.74; 1.2]	0.74 [0.62; 1.0]*	0.97 [0.69; 1.72]†	0.0158; 0.0058^K^
Haemoglobin (g/dL)	13.2 [12.1; 14.48]	12.2 [10.9; 13.60]*	12 [10.75; 12.93]*	0.0011; 0.0002^K^
Haematocrit (%)	38.95 [35.68; 41.78]	36.9 [32.3; 39.60]*	36.35 [33.33; 38.83]*	0.0120; 0.0133^K^
Leucocytes (x1000/mm^3^)	7.43 [5.54; 11.45]	7.69 [5.40; 9.87]	8.77 [7.01;13.25]	0.0671^K^
Lymphocytes (x1000/mm^3^)	1.06 [0.76; 1.38]	1.06 [0.75; 1.46]	0.91 [0.7; 1.21]	0.3952^K^
Neutrophils (x1000/mm3)	5.68 [4.08; 9.24]	5.95 [4.08; 7.93]	7.21 [5.61; 11.2]†	0.0206^K^
Platelets (x10^3^/μL)	227.5 [177.8; 317.3]	217 [170.5; 282.5]	247.5 [199.8; 314]	0.1938^K^
Cardiovascular disease (%)	21	15	11	0.1470^C^
Hypertension	48	51	67	0.0147^C^
Obesity (%)	38	36	54	0.0187^C^
Diabetes (%)	23	31	57	<0.0001^C^
Psychiatric diseases (%)	1	3	2	0.6004^C^
Asthma (%)	0	6	2	0.0274^C^
COPD (%)	5	2	2	0.3567^C^
Intubation (%)	32	25	78	<0.0001^C^
Length of intubation (days)	0 [0; 2]	0 [0; 0]	5.5 [0; 9.75]*^,^†	<0.0001; <0.0001^C^
Length of hospital stay (days)	8 [5.5; 15]	9 [5; 18.5]	21.5 [12; 32.25]*^,^†	<0.0001; <0.0001^C^

CRP, C reactive protein; AST, aspartate aminotransferase; ALT, alanine aminotransferase; COPD, chronic obstructive pulmonary disease; * vs. Control; † vs. SR; ^/^ Number of patients per group: 27, 28, 24; ^//^ Number of patients per group: 61, 71, 53; ^///^Number of patients per group: 66, 75, 48; ^A^One-way ANOVA followed by Tukey post-test; ^K^Kruskal-Wallis test followed by Dunn’s test; ^C^Chi-square test. SR, Self-Reported Symptoms group; SRPF, Self-Reported Symptoms and decreased Pulmonary Function group.

**Figure 2:**
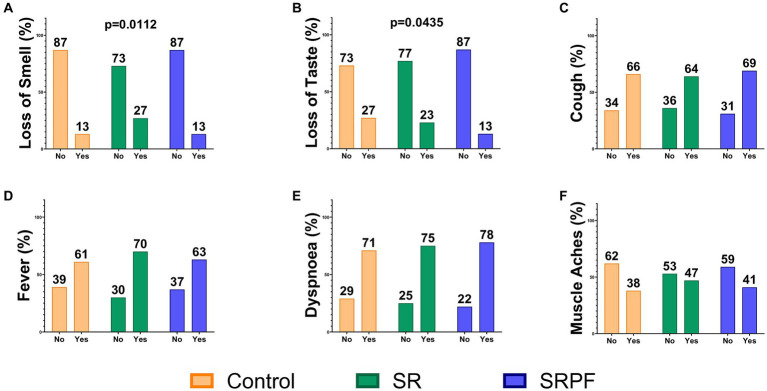
Signs and symptoms at hospital admission. Data are presented as the percentage of patients in the group. **(A)** Loss of Smell; **(B)** Loss of Taste; **(C)** Cough; **(D)** Fever; **(E)** Dyspnoea; **(F)** Muscle Aches. Chi-squared test was used to compare frequencies. SR, Self-Reported Symptoms group; SRPF, Self-Reported Symptoms and decreased Pulmonary Function group.

COVID-19 signs and symptoms are presented in Figure 2 and Supplementary Figure 1: SR showed higher frequency of anosmia (27%) at hospital admission, compared to the Control (13%) and SRPF (13%). Impaired sense of taste was a less frequent symptom in SRPF (13%) in relation to the Control (27%) and SR (23%) groups.

### 3.2 Follow-up assessment

#### 3.2.1 General parameters

After ≅ 6 to 12 months (median of 202 days) of hospital discharge, patients who were hospitalised in 2020, during the first wave of COVID-19 in Brazil, were invited back to Hospital das Clínicas to go through follow-up assessment, which included blood tests, questionnaires, and physical evaluation. Months from hospitalisation to the follow-up assessment are shown in Supplementary Figure 2. Higher serum CRP was found in SR and SRPF, compared with the Control (*q =* 0.010 and *q =* 0.007, respectively), and higher glycated haemoglobin content was found for SRPF, compared with Control and SR (*q =* 0.0003 and *q =* 0.007, respectively). SR and SRPF showed lower values for haemoglobin compared with the Control group (*q =* 0.010 and *q =* 0.007, respectively), as shown in Table 2. Lymphocyte count was higher in SR and SRPF compared to Control group (*q =* 0.0485 and *q =* 0.0013, respectively).

**Table 2:** General parameters in the follow-up assessment.

	Control	SR	SRPF	Dunn’s adjusted value of *p*	Dunn’s *q* value
Haemoglobin (g/dL)	14.33 [12.89; 15.42]	13.27 [12.08; 14.41]*	13.02 [11.93; 14.27]*	0.0692; 0.0253	0.0100; 0.0070
Glycate haemoglobin (mmol/mol)	5.57 [5.20; 5.80]	5.77 [5.19; 6.28]	6.24 [5.65; 8.12]*,^†^	0.0003; 0.0338	0.0003; 0.0074
Lymphocytes (x1000/mm^3^)	1.89 [1.54; 2.35]	2.01 [1.56; 2.47]*	2.27 [1.78; 2.97]*	0.3877; 0.0035	0.0485; 0.0013
Platelets (×10^3^/μL)	242.35 [197.78; 286.95]	259.10 [213.04; 303.55]	264.00 [219.35; 316.08]	n.s.	n.s.
CRP (mg/dL)	3.96 [1.40; 6.98]	5.77 [2.82; 10.45]*	6.08 [3.70; 10.33]*	0.0692; 0.0422	0.0100; 0.0074
D-dimer (mg/L)	410.02 [257.51; 580.05]	418.01 [228.75; 733.17]	463.00 [314.00; 720.00]	n.s.	n.s.

Comparisons were performed using the Kruskal-Wallis test followed by Dunn’s *post-hoc* test after adjustment by days to follow-up assessment. Statistical significance was considered when *q* < 0.052 in the Dunn’s *post-hoc* test. n.s. non-significant in Kruskall-Wallis test, *post-hoc* test not performed. * vs. Control; † vs. SR; CRP, C-reactive protein; SR, Self-Reported Symptoms group; SRPF, Self-Reported Symptoms and decreased Pulmonary Function group.

#### 3.2.2 Self-reported symptoms

SR and SRPF presented several persistent and/or newly acquired symptoms related to PCC at the time of the follow-up assessment, which are shown in Figure 1 and Supplementary Figure 3.

#### 3.2.3 Serum cytokines, chemokines and growth factors

**Figure 3:**
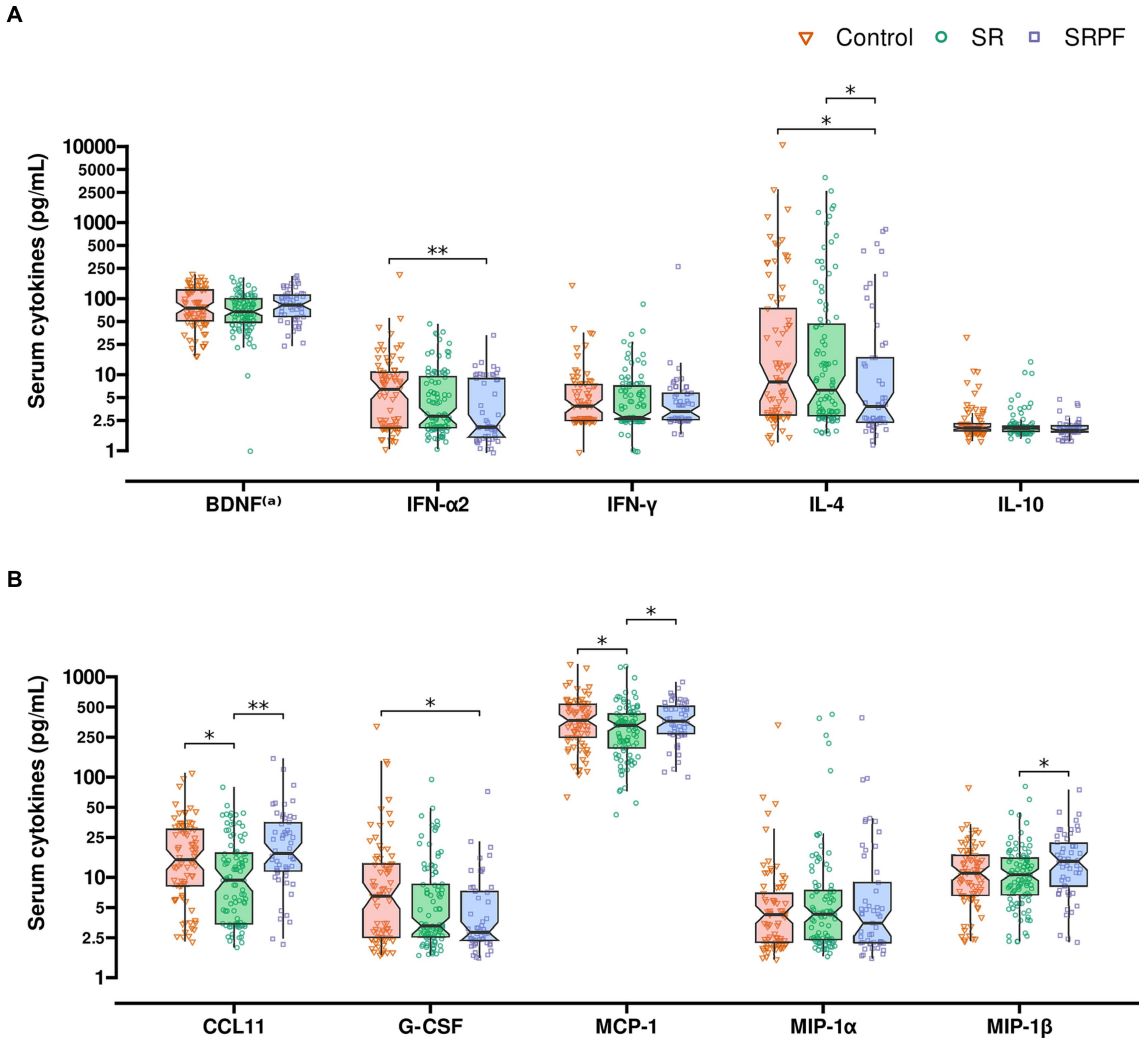
Serum cytokines. **(A)** Interferons and interleukins. **(B)** Growth factors and chemokines. Comparisons were performed using the Kruskal-Wallis test followed by Dunn’s *post-hoc* test after adjustment by days to follow-up assessment. *q value <0.052; ** adjusted value of *p* < 0.05. **(a)** Serum BDNF unit: ng/dL. BDNF brain-derived neurotrophic factor; CCL11 C-C motif chemokine ligand 11 (eotaxin-1); G-CSF granulocyte colony-stimulating factor; IFN interferon; IL interleukin MCP1 monocyte chemoattractant protein 1; MIP macrophage inflammatory protein; SR, Self-Reported Symptoms group; SRPF, Self-Reported Symptoms and decreased Pulmonary Function group.

The analysis of serum cytokines demonstrated SRPF to present lower IFN-α2 concentration in the circulation, as compared with the Control group (*q =* 0.007). Serum IL-4 was lower in SRPF vs. Control (*q =* 0.018) and SR (*q =* 0.030). Several differences were noted in regard to chemokines: serum CCL11/eotaxin-1 concentration was lower in SR, compared with both other groups (*q =* 0.012 vs. Control and *q =* 0.001 vs. SRPF). SRPF presented lower G-CSF content compared with the Control (*q =* 0.014). Moreover, SRPF presented a higher MIP-1β concentration in the circulation, in relation to SR (*q =* 0.029). SR showed lower MCP-1 serum levels, compared with both other groups (*q* = 0.052 for both groups). These results are presented in Figure 3. The full set of results are shown in Supplementary Table 1.

#### 3.2.4 Muscle function

**Figure 4:**
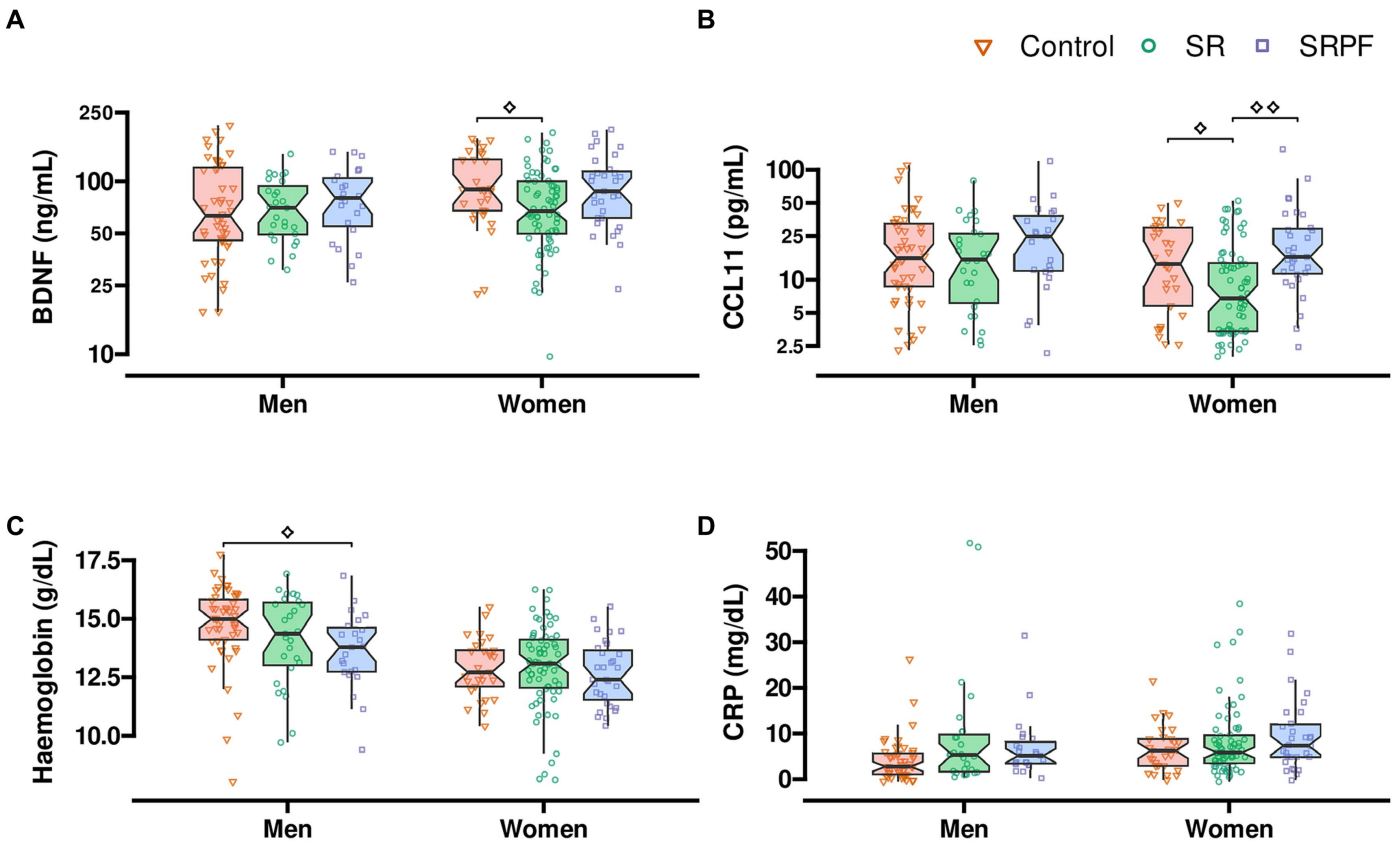
Content of BDNF, CCL11, haemoglobin, and lymphocyte count after sex stratification. **(A)** Serum BDNF. **(B)** Serum CCL11. **(C)** Blood haemoglobin. **(D)** Serum CRP. Comparisons were performed using the Kruskal-Wallis test followed by Dunn’s *post-hoc* test after adjustment by days to follow-up assessment. ⟡, ⟡⟡*q* value <0.063; ⟡⟡adjusted value of *p* < 0.05. BDNF brain-derived neurotrophic factor; CCL11 C-C motif chemokine ligand 11 (eotaxin-1); CRP, C-reactive protein; SR, Self-Reported Symptoms group; SRFF, Self-Reported Symptoms and decreased Pulmonary Function group.

Women in SR presented lower grip strength, when compared with women of the SRPF groups (*q =* 0.002), as presented in Table 3. In the timed up-and-go test, men in SR and SRPF took longer to complete the test, compared with men in the Control group (*q =* 0.059 and *q =* 0.003, respectively). The Borg dyspnoea scale is a function test in which the participants report employing a numerical score on the sensation of dyspnoea before and after a simple physical exercise test. In this case, patients were asked to sit and stand from a chair as many times as possible for 1 min. Before the test, SRPF male patients reported higher dyspnoea compared with male patients in the Control and SR (*q =* 0.002 and *q =* 0.063, respectively). Male patients in SRPF performed a lower number of test repetitions than male patients enrolled in the Control and SR groups (*q =* 0.002 and *q =* 0.063, respectively), as shown in Table 3. CCL11 concentration was lower in SR female patients, vs. Control and SRPF female patients (*q* = 0.055 and *q* = 0.002, respectively). BDNF serum content was lower in females in SR, compared with Control female patients (*q =* 0.055; Figure 4). All the stratified parameters are shown in Supplementary Table 2.

**Table 3:** Physical performance in the follow-up assessment stratified by sex after adjustment by the follow-up interval.

	Control	SR	SRPF	Dunn’s adjusted value of *p*	Dunn’s *q* value
*Handgrip (kgf)*					
Men	32.43 [27.22; 43.47]	34.66 [24.69; 37.98]	27.83 [24.57; 31.05]	n.s.	n.s.
Women	16.54 [8.50; 22.85]	10.09 [4.89; 18.37]	18.13 [15.77; 20.35]^†^	0.0082	0.0019
*Up-and-go test (sec)*					
Men	10.62 [9.53; 12.24]	12.40 [11.07; 14.02]*	13.31 [12.30; 15.97]*	0.4205; 0.0133	0.0591; 0.0027
Women	12.96 [10.69; 14.30]	13.00 [11.24; 15.89]	13.04 [11.95; 16.29]	n.s.	n.s.
*Borg dyspnoea scale, pre-test*					
Men	0.06 [-0.03; 0.30]	0.10 [-0.01; 0.96]	1.98 [0.64; 2.92]*,^†^	0.0062; 0.4592	0.0018; 0.0633
Women	0.28 [0.08; 1.95]	1.22 [0.07; 3.02]	1.21 [0.14; 3.03]	n.s.	n.s.
*Borg dyspnoea scale, post-test difference (bpm)*					
Men	1.99 [0.47; 2.97]	3.01 [1.00; 3.31]	1.97 [1.08; 2.10]	n.s.	n.s.
Women	1.56 [0.48; 2.17]	1.95 [0.49; 3.00]	1.56 [0.98; 2.33]	n.s.	n.s.
*Peripheral oxygen saturation, basal (%)*					
Men	97.28 [95.61; 98.16]	96.41 [94.66; 97.44]	96.80 [95.66; 97.82]	n.s.	n.s.
Women	97.06 [94.93; 97.84]	97.32 [96.31; 98.08]	96.59 [94.99; 97.67]	n.s.	n.s.
*Peripheral oxygen saturation, post-test difference (%)*					
Men	0.42 [-0.11; 2.03]	0.01 [-1.01; 1.99]	0.07 [-0.89; 1.04]	n.s.	n.s.
Women	0.72 [0.01; 1.22]	-0.00 [-0.92; 0.83]	-0.05 [-0.99; 1.24]	n.s.	n.s.
*Test repetitions (n)*					
Men	21.39 [18.51; 25.77]	19.03 [14.15; 24.44]	15.63 [12.16; 18.47]*,^†^	0.0018; 0.4568	0.0018; 0.0632
Women	18.91 [13.87; 23.33]	14.45 [12.01; 18.77]	13.60 [10.33; 16.27]	n.s.	n.s.

Comparisons were performed using the Kruskal-Wallis test followed by Dunn’s *post-hoc* test after adjustment by days to follow-up assessment. Statistical significance was considered when *q* < 0.063 in the Dunn’s *post-hoc* test. n.s. non-significant in Kruskall-Wallis test, *post-hoc* test not performed. * vs. Control; † vs. SR; SR, Self-Reported Symptoms; SRPF, Self-Reported Symptoms and decreased Pulmonary Function group.

#### 3.2.5 Multiple linear regression

A multiple linear regression adjusted for age, sex and follow-up interval showed that the profile of serum cytokine levels moderately predicts the total number of self-reported symptoms observed (*R*^2^ = 0.20), with a partial *F* test of 3.54 (*p* = 0.00002), as presented in Table 4. IFN-α2 and the number of symptoms showed a negative association (*p* = 0.023, *β* = −2.43, CI 95% [−4.78; −0.23]). Therefore, low IFN-α2 is present in participants with more self-reported symptoms. Another multiple linear regression, this time stratified by sex, and with cytokine levels adjusted for age, showed that in women, cytokines also relate with the variation in handgrip strength (*R*^2^ = 0.24), with a partial *F* test of 3.73 (*p* = 0.015), as shown in Supplementary Table 3. Handgrip strength was positively associated with TNFα (*p* = 0.039, *β* = 11.87). In men, cytokine levels were not predictive of handgrip strength. The timed up-and-go test and the number of test repetitions performed in the Borg Scale test did not show a significant association with cytokine levels, either.

**Table 4:** Multiple linear regression to test the ability of serum cytokines in predicting the number of self-reported symptoms.

Cytokine	*β*	Value of *p*
Interval to follow-up	−0.01	0.248
Age	−0.03	0.276
**Sex (male)**	−**3.40**	**0.00005**
BDNF	−2.42	0.092
EGF	−0.09	0.921
CCL11	−0.17	0.878
**IFN-α2**	−**2.43**	**0.023**
IL-8	1.10	0.194
IL-10	−2.34	0.307
IL-17	−0.47	0.839
IP-10	2.90	0.087
MCP-1	−1.49	0.381
MIP-1α	−0.13	0.874
MIP-1β	0.65	0.624
TNF-α	1.76	0.420

The confounding factors age, sex, and follow-up interval were also included in the model. *R*^2^ = 0.20, *F* (15) = 3.54, *p* = 1.9 × 10^-5^. BDNF – ng/dL, other cytokines – pg/dL.

## 4 Discussion

This is the first study, to the best of our knowledge, to examine inflammation related to PCC in a large sample of patients (*n* = 227) who presented several symptoms and lower muscle function as compared with controls (patients who were hospitalised with COVID-19 in 2020 but presented a low frequency of self-reported symptoms 6–12 months after discharge). SRPF presented pulmonary lesions together with the presence of several alterations, also reported by SR. Although tissue and organ damage may contribute to sustained inflammation, both groups presented greater levels of CRP and disrupted cytokine profile at the follow-up assessment, disregarding the absence of diagnosed lesions in SR. Persistent inflammation in PCC was accompanied by diminished physical performance, when compared with controls. These alterations were still detectable after considering the differences in follow-up interval in the statistical analyses.

The presence of comorbidities has been linked to COVID-19 severity and may be related to PCC, as well (25). Following that assumption, SRPF presented a higher frequency of patients with hypertension, obesity, and diabetes, as assessed at hospital admission. SRPF also required respiratory support more often than the other groups, and intubation during hospital stay was of greater length in this group when compared with the others.

Insulin resistance and stress-related hyperglycaemia are quite common in patients with severe acute illnesses (26). The presence of diabetes is related to acute disease severity (27). Diabetes was more frequent in SRPF (57%), followed by SR (31%) and then, by the Control group (23%). In agreement with that, SRPF showed higher glycated haemoglobin concentration in the follow-up measurements. Diabetes mellitus is characterised by chronic inflammation (28), which may have contributed to higher acute disease severity during hospitalisation and increased systemic inflammation in the follow-up shown by SRPF.

SR and SRPF showed persistent inflammatory dysfunction, with higher levels of CRP detected also in the follow-up assessment. Changes in CRP, D-dimer levels and lymphocyte count have been reported as biomarkers of PCC (29). The SRPF group presented lower INF-α2 serum content, compared with the Control group. Furthermore, lower INF-α2 was associated with the frequency of self-reported symptoms in the follow-up. Lower levels of IFN-α2 have been associated with COVID-19 severity during acute infection (30). Interferons are important mediators of antiviral response and IFN-α2 is frequently employed in the treatment of viral infections. Type I interferons bind to a ubiquitously expressed membrane receptor composed of IFNAR1 and IFNAR2 subunits and activate its signalling cascade in virtually all cell types (31). MCP-1, an IFN-γ inducible chemokine, was altered in patients with PCC. MCP-1 was lower in SR compared to the other groups.

A dysregulation in cytokine content has been linked to T cell exhaustion in patients who recovered from COVID-19 (*n* = 39) (32), with higher levels of IL-1β, IL-1RA, IL-7, IL-8, IL-10, IFN-γ, and MIP-1α, and a decrease in IL-9, CCL11, MIP-1β, and RANTES, as compared with healthy controls (*n* = 43) (32). In another study, patients who were followed during acute COVID-19 were also monitored after a median of 219 days post-infection (33). Patients with PCC (*n* = 12) presented high serum INF-α, TNFα, G-CSF, IL-1β, IL-6, IL-13, IL-17A, and low IP-10, when compared with healthy controls and showed persistence of dysregulated immune response after acute-infection (33, 34).

Phetsouphanh et al. (35) compared patients with PCC to individuals who recovered from COVID-19 and did not complain of any PCC-related symptoms, and with patients who were exposed to other coronaviruses and with healthy controls. After 4 months of infection, patients with PCC and patients without PCC-related symptoms did not differ in regard to the concentration of circulating cytokines, but both groups presented higher levels of circulating IFN-α, IFN-λ1, IP-10 and IL-8, in relation to the other groups (healthy controls and volunteers exposed to other coronaviruses) (35). Furthermore, there was a decrease in these cytokines’ concentration after 8 months, in both groups that had been exposed to SARS-CoV-2 (35). Another study that compared PCC patients with patients who had COVID-19 but did not present PCC, and patients who had PCC and recovered, showed that ongoing PCC could be characterised by higher circulating IL-1β, IL-6 and TNFα, 8 to 10 months after acute infection (36). The authors suggest that PCC is caused by a reprogramming of pro-inflammatory immune cells (36).

Sexual dimorphism in immune response seems to influence acute COVID-19 severity, as male patients presented higher innate immune response-related cytokine levels in acute infection, while female patients showed higher type I interferon response during acute disease course (37). A study with 213 patients who recovered from COVID-19 reported that male and female patients presented no difference in the prevalence of chronic symptoms (lasting more than 3 months after infection), despite female patients showing higher frequency of acute symptoms such as dyspnoea, headache, myalgia, and palpitations, and male patients, higher frequency of acute respiratory failure and requirement for ventilation (38). Our results indicate that SRPF females presented higher inflammatory response, with higher CCL11 concentration. Both male and female patients with PCC showed poorer results in physical performance tests than those of control patients. We have previously reported that patients with PCC symptoms presented a higher frequency of physical inactivity and that this frequency varied based on the number of PCC symptoms (17). Particularly, the presence of dyspnoea, fatigue, insomnia, post-traumatic stress, and severe muscle/joint pain was able to predict physical inactivity in 614 patients (17).

Patients with PCC, parosmia, and loss of taste described also neuropsychiatric alterations, which included lower memory perception and lower episodic memory, indicating that cognitive dysfunction was present, as showed by Damiano et al. (39) (same cohort). Furthermore, the presence of PCC was mainly distinguished by fatigue, psychiatric dysfunction and cognitive alterations and related to higher levels of CRP and D-dimer in this same population (16). CCL11/eotaxin is a chemokine responsible for attracting eosinophils to the affected tissues. High levels of this chemokine can repress neurogenesis and promote cognitive impairment (40). It has been reported that patients with brain fog after COVID-19 infection exhibited higher levels of CCL11/eotaxin in the plasma, when compared with patients who had COVID-19 and did not show signs of this symptom (41). A multivariate analysis showed that the presence of autoimmune disease and male sex contributed to this difference (41). In this same study, CCL11/eotaxin was still high in mouse cerebrospinal fluid 7 weeks after SARS-CoV-2 infection. On the other hand, plasma CCL11/eotaxin, which was high 7 days post-infection was normalised after 7 weeks (41).

BDNF is a neurotrophin family member that is involved with processes of synaptic plasticity related to learning, memory, and behaviour (42). BDNF participates on the differentiation and maturation of neurotransmitter systems such as stress, reward, and motivation systems in the brain (42). Furthermore, lower levels of BDNF were associated with neuronal atrophy and reduced synaptic plasticity (43). BDNF levels are lower in patients with depression who were not taking specific medication, compared with healthy volunteers and in patients with depression under antidepressant treatment (44).

Mechanical ventilation and immobilisation have devastating consequences for skeletal muscle mass (45). In acute disease, patients’ lower muscle function was found to be associated with higher mortality (46). In our study, fatigue, dyspnoea, muscle and joint pain, persistent weakness and walking impairment were highly prevalent in individuals with PCC. Also, these patients presented lower muscle function with reduced handgrip strength, were slower in the up-and-go test and exhibited lower performance in the dyspnoea test. These results indicate that self-reported symptoms occurred together with debilitating alterations in individuals with PCC.

Sarcopenia has been associated with fatigue in patients with PCC, and longer hospital stay has been related to higher risk of developing sarcopenia after hospital discharge (47). Changes in muscle architecture in patients with PCC and fatigue were also reported (48). Furthermore, respiratory muscle dysfunction has been associated with a decrease in inspiratory muscle strength and higher neuroventilatory activity, which reflects in lower muscle functional performance in the six-minute walking test (49). Respiratory muscle training, strength training, and/or aerobic exercise, especially low-intensity exercise have been proposed as interventions for PCC, possibly leading to greater physical fitness and improved tolerance to physical effort (50).

Muscle dysfunction and myalgias have been described in patients with SARS (51, 52). Patients with the moderate and severe forms of the acute disease presented a 32% reduction in grip strength and 13% reduction in the six-minute walking distance, as well as occupational impact, with only 40% of patients returning to work 2 to 3 months after hospital discharge (51). SARS-CoV1 infection also caused muscle atrophy, myofibers disarrangement (51) and neuronal demyelination (52). Moreover, a 5-year prospective evaluation of long-term outcomes of patients recovered from Acute Respiratory Distress Syndrome (ARDS) showed that young individuals presented persistent physical activity limitations and lower quality of life, albeit showing close to normal pulmonary function (53). These individuals also presented higher health care requirements, causing ARDS-related symptoms to be envisaged as a disability (53). ARDS Survivors had lost 18% of their body weight at ICU discharge (54) and weight regain in the following year was mainly due to fat mass accretion (55). These patients also presented muscle weakness and fatigue, the likely cause of their observed functional limitations (54). Deleterious changes in body composition, such as loss of skeletal muscle mass and increased adiposity, which were observed in patients with ARDS (55), can lead to a higher risk of developing other chronic diseases (56, 57).

Our PCC study population was composed of two distinguishable groups; we separated patients with pulmonary lesions from patients without lung damage to better evaluate the changes in inflammatory markers. Patients in SR were very similar to those in SRPF regarding self-related symptoms, such as fatigue. However, serum cytokines in the SRPF group displayed a more pro-inflammatory profile than that of SR, which may indicate that organ damage contributes to the persistent inflammation or even that the tissue damage is a result of one such sustained inflammation. Furthermore, SR can be distinguished by the higher prevalence of loss of smell at hospitalisation (27% vs. 13% in the other two groups), higher loss of appetite, headaches, and hearing loss in the follow-up. These findings may point out the possible prediction of PCC development. PCC has remarkable similarities to post-sepsis syndrome, as these two conditions are characterised by persistent symptoms after the acute disease, with a decrease in the quality of life (58, 59). In both, the long-term sequelae seem related to inflammatory dysregulation (58). Sepsis survivors present a higher risk of death in the subsequent years (59). Similarly, it has been demonstrated that individuals who were hospitalised due to SARS-CoV-2 infection presented a higher risk of death 2 years after the acute infection (60).

Exacerbated inflammation has been linked to severe acute SARS-Cov-2 infection (61). The virus, in this context, seems to be able to evade the innate immune response, replicating and provoking a potent pro-inflammatory response, with immune cell infiltration to the lungs, causing tissue damage (30, 62). Following that, secretion of potent pro-inflammatory cytokines activates coagulating cascades and hence, propagates the effects of the disease to several organs (62). PCC physiopathology has been postulated to involve autoimmunity, together with immune dysregulation, viral tissue remnants, tissue damage, dysbiosis (63), endothelial dysfunction and blood clotting, and altered neurological signalling (64).

Body weight loss, higher CRP and D-dimer and lower performance in the 1 min sit-to-stand and handgrip strength tests, evaluated months after hospital discharge, were previously associated with latent trait’s severity estimated by the Item Response Theory (IRT) in 749 patients that were hospitalised due to COVID-19 (16). These results indicate that objective measurements, such as systemic inflammation, were related to the co-occurrence of fatigue, psychiatric and cognitive manifestations (16). In the present study we were able to demonstrate an inverse association between IFN-α2 and the number of self-reported PCC symptoms, further extending the role of inflammation in this condition.

It is important to state the limitations of our work. There was a considerable interval in the follow-up assessment, therefore, we performed an adjustment in the statistical analysis to overcome this limitation. We could not assess cytokines and chemokines during the hospitalisation period. Muscle function was not evaluated at hospital admission. We were able to show, however, differences between individuals who recovered from COVID-19 and present persistent distinct PCC-related symptoms. The sample size was not pre-specified what may limit generalisation of findings, also this is a single-centre study, in which all the participants were hospitalised due to acute infection; we performed retrospective allocation of participants to groups; and a high number of multiple comparisons. The strengths of this study include the robust sample size; the group categorisation through symptoms, which helps to understand the variability of PCC; the gender-specific analysis addressing the differences of PCC in men and women; and the inclusion of a Control group composed of participants that recovered from COVID-19 and did not develop PCC.

This study was able to unravel specific inflammatory markers associated with different symptom profiles, representing a contribution to the development of targeted interventions and personalised treatments. We also demonstrated that men and women suffering from PCC must be considered separately. Therefore, the variability in PCC must be taken in regard together with sex-specific differences.

Disrupted inflammatory markers in combination with persistent fatigue – among other self-referred symptoms – and lower muscle function, are important alterations observed in individuals with PCC. Here we have shown that different PCC profiles can be distinguished through their circulating inflammatory markers. Additionally, our findings have demonstrated the heterogeneity of PCC manifestations. A precise treatment approach will require evaluation of biomarkers, together with organ damage diagnosis and self-related symptom assessment. PCC is a debilitating syndrome that demands further attention and lower muscle function must be considered as an important sequela that can have long-lasting consequences.

## Data availability statement

The raw data supporting the conclusions of this article will be made available by the authors, without undue reservation.

## Ethics statement

The studies involving humans were approved by Comitê de Ética do Hospital das Clinicas da Faculdade de Medicina da USP. The studies were conducted in accordance with the local legislation and institutional requirements. The participants provided their written informed consent to participate in this study.

## Author contributions

GC: Data curation, Formal analysis, Investigation, Writing – original draft, Writing – review & editing. LG: Data curation, Formal analysis, Writing – original draft, Writing – review & editing. AFR: Conceptualization, Investigation, Methodology, Writing – original draft, Writing – review & editing. GS: Formal analysis, Writing – review & editing, Data curation. AT: Data curation, Formal analysis, Writing – review & editing. EC-N: Conceptualization, Project administration, Resources, Writing – review & editing. HS: Conceptualization, Project administration, Resources, Writing – review & editing. SM: Conceptualization, Project administration, Resources, Writing – review & editing. LT: Conceptualization, Project administration, Writing – review & editing. VC: Conceptualization, Writing – review & editing. JK: Conceptualization, Writing – review & editing. AA: Data curation, Project administration, Writing – review & editing. APR: Data curation, Project administration, Writing – review & editing. ARB: Data curation, Formal analysis, Writing – review & editing. AS: Data curation, Formal analysis, Writing – review & editing. APB: Data curation, Writing – review & editing. MSa: Data curation, Project administration, Writing – review & editing. CL: Data curation, Project administration, Writing – review & editing. BB: Data curation, Project administration, Writing – review & editing. CC: Data curation, Project administration, Writing – review & editing. LK: Data curation, Project administration, Writing – review & editing. RD: Data curation, Writing – review & editing. MI: Data curation, Formal analysis, Project administration, Writing – review & editing. JR: Data curation, Resources, Writing – review & editing. FL: Data curation, Resources, Writing – review & editing. JO: Writing – review & editing. EM: Data curation, Project administration, Resources, Writing – review & editing. LB: Conceptualization, Data curation, Project administration, Resources, Writing – review & editing. OF: Conceptualization, Data curation, Project administration, Resources, Writing – review & editing. GB: Conceptualization, Data curation, Investigation, Project administration, Resources, Writing – review & editing. MSe: Conceptualization, Funding–acquisition, Project administration, Resources, Supervision, Writing – original draft, Writing – review & editing.

## Footnotes

^1^ https://ensaiosclinicos.gov.br/rg/RBR-8z7v5wc
