# Supplementary material for: Post-COVID-19 condition: systemic inflammation and low functional exercise capacity
Source: Front Nutr. 2024 Mar 14;11:1295026. doi: 10.3389/fnut.2024.1295026 (PMC10973152; doi:10.3389/fnut.2024.1295026)
Supplement: Supplementary file 1 [file Data_Sheet_1.pdf]

Supplementary Material

1 Methods

Acute COVID-19 during hospitalisation was confirmed by RT-PCR performed with the equipment Abbott m200RT (Abbott Laboratories, Chicago, Illinois, USA) at the Central Laboratory Division of Hospital das Clínicas, São Paulo, Brazil. For highly suspect cases with at least 2 RT-PCR negative tests, serum antibodies were measured by chemiluminescent immunoassays on Liaison XL analyser (DiaSorin S.p.A., Saluggia, Italy) performed after seven days of the onset of symptoms or in subjects with high clinical suspicion for whom a RT-PCR test was not available up to the 10th day of symptom beginning.

1.1 Statistical Analysis

1.1.1 Cytokine Detection Limit

Readings from the cytokine panel reported a fraction of levels below detection threshold between 0% and 100% (median of 46%), characterizing a left-censored distribution for most cytokines. For observations with censored values, a value equal to the mean detection limit value for that cytokine divided by the square root of 2 was assigned.

1.1.2 Batch Effect, Systemic Bias, and Variable Reduction

All numerical data were corrected for systematic bias, including variation in the time interval, in days, between hospital discharge and follow-up assessment. The cytokine panel was divided into 5 batches, with batches 1 to 3 carrying out similar number of samples for each group and batches 4 and 5 presenting fewer SRPF samples. The nonparametric version of the ComBat method (1) was used to correct for batch effects. After that, all tested variables were corrected for interval to follow-up by fitting a linear regression model.

As we had many variables to test hypotheses over, exclusion or combination of redundant or highly correlated variables was performed, as to increase the statistical power of the analysis. In particular, the cytokines IL-1α, IL-5, IL-6 and IL-13 were excluded in favour of IL-4 and TNF-β, which showed higher detection levels and were all positively correlated. Similarly, IL-1RA, IL-12p40 and IL-15 were excluded in favour of IL-7, IFN-α2, IFN-γ, G-CSF and VEGF, all with high positive correlations.

For blood laboratorial tests, leukocytes count was excluded in favour of lymphocytes count, haematocrit in favour of haemoglobin concentration, and serum iron in favour of ferritin serum content. For the function tests, systolic and diastolic blood pressures were combined within a mean blood pressure variable, while the highest measurement of both arms was chosen to represent the handgrip test result.

2 Results

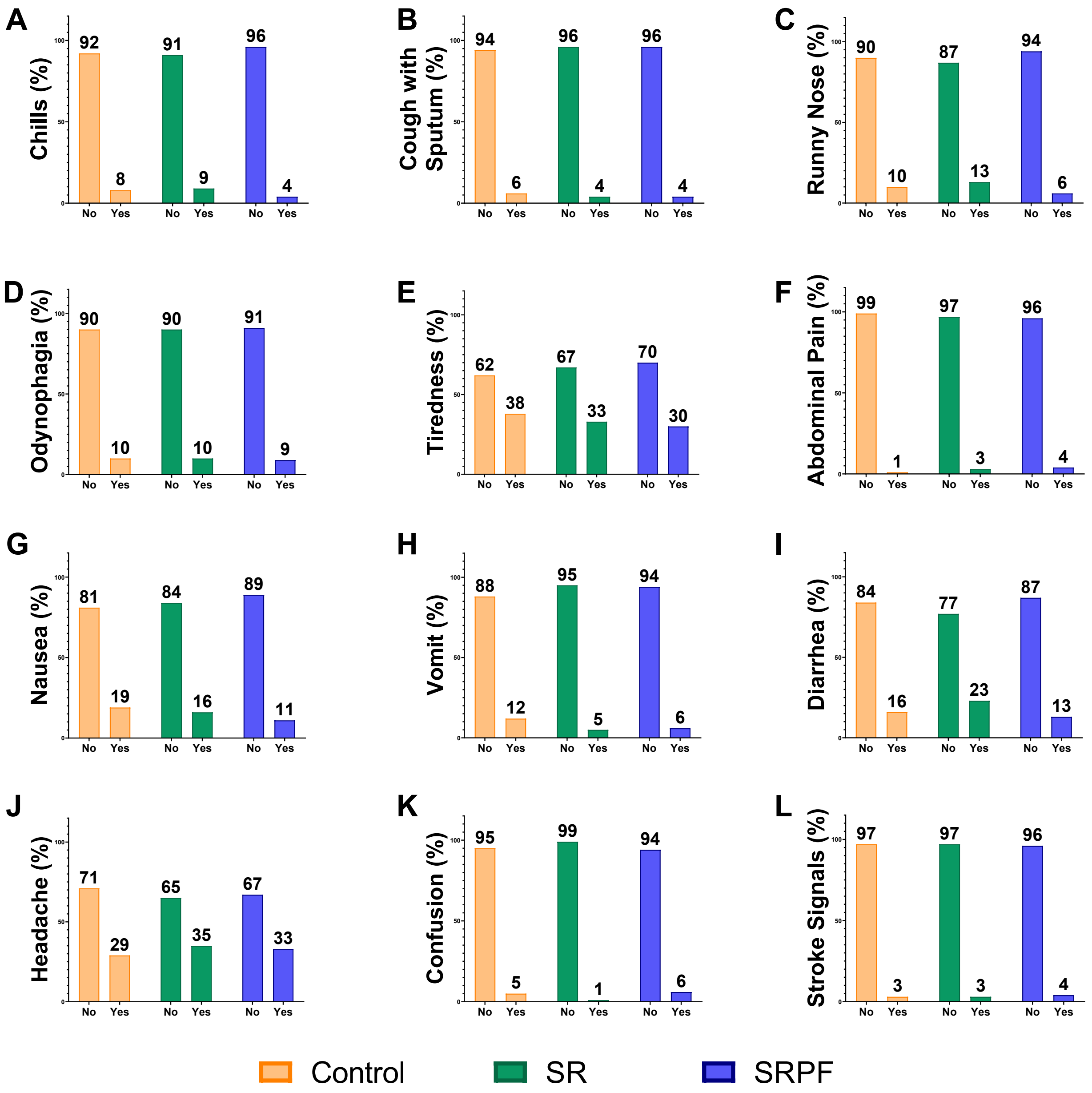

Supplementary Figure 1. Signs and symptoms at hospital admission. Data are presented as the percentage of patients in the group. A. Chills; B. Cough with Sputum; C. Runny Nose; D. Odynophagia; E. Tiredness; F. Abdominal Pain; G. Nausea; H. Vomit; I. Diarrhoea; J. Headache; K. Confusion; L. Stroke Signals. Chi-square test was used to compare frequencies. SR – self-reported symptoms group; SRPF – self-reported symptoms and decreased pulmonary function group.

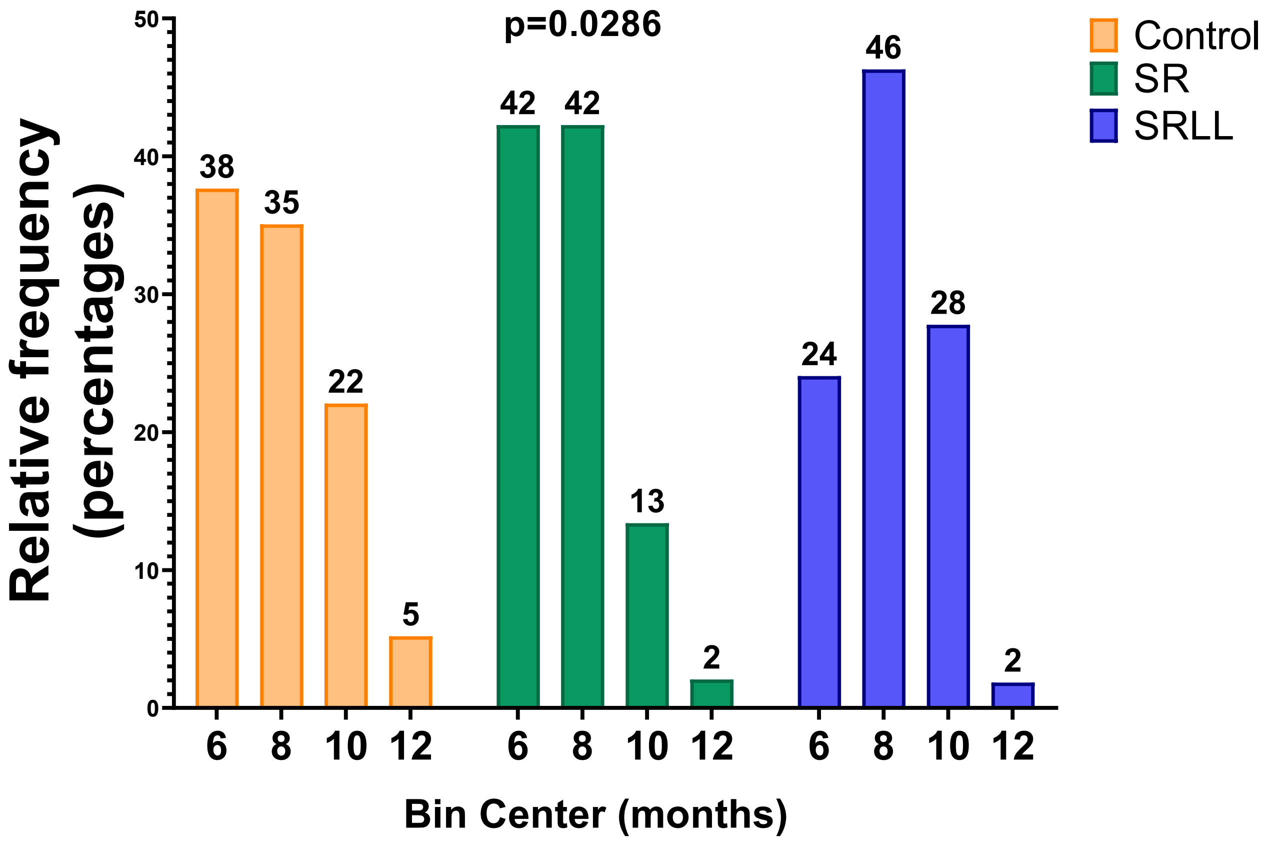

Supplementary Figure 2. Distribution of months to follow-up assessment. Chi-square test was used to compare frequencies. SR – self-reported symptoms group; SRPF – self-reported symptoms and decreased pulmonary function group.

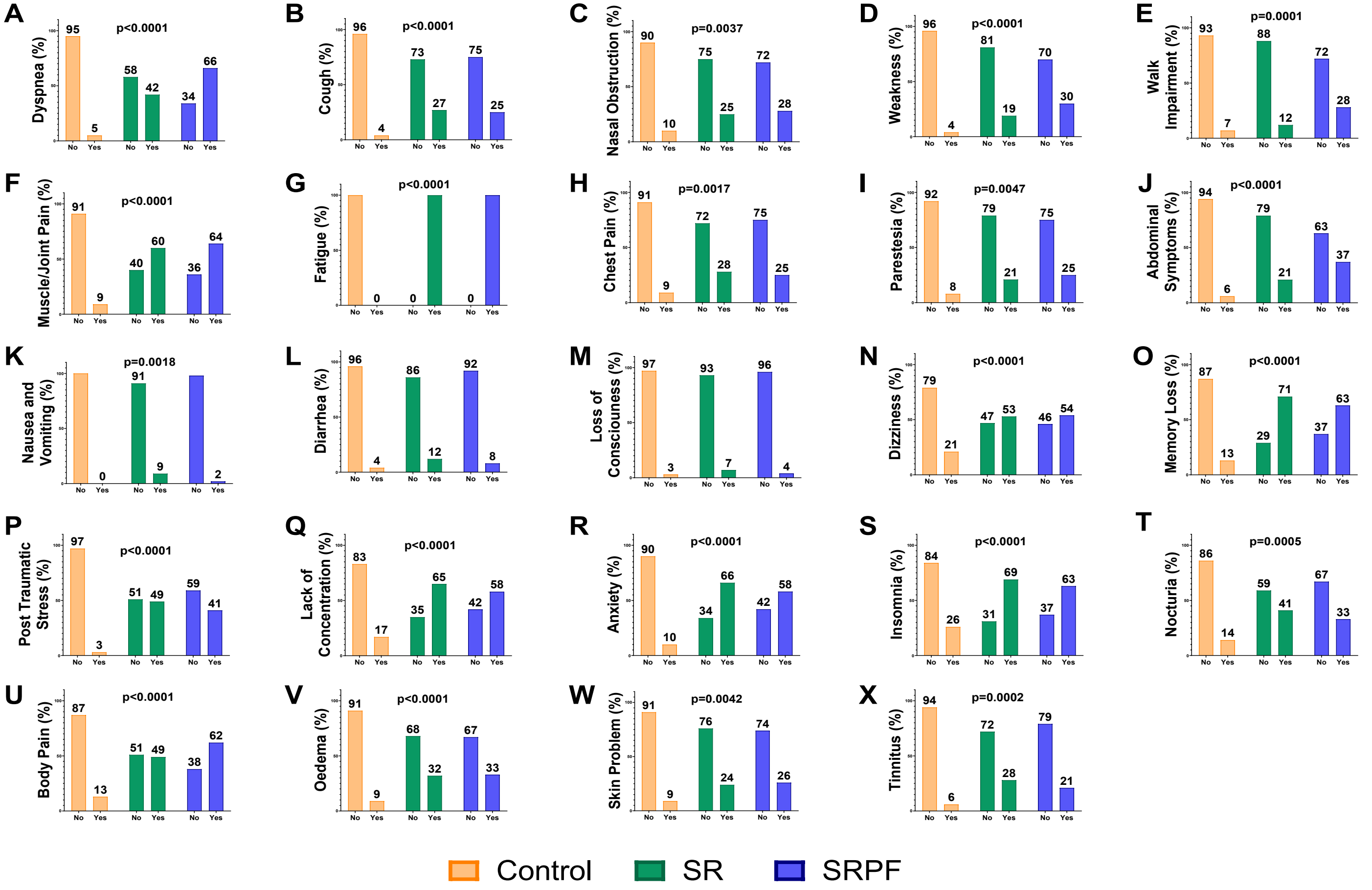

Supplementary Figure 3. Signs and symptoms in the follow-up assessment. Data are presented as the percentage of patients in the group. A. Dyspnea; B. Cough; C. Nasal Obstruction; D. Weakness; E. Walk Impairment; F. Muscle/Joint Pain; G. Fatigue; H. Chest Pain; I. Paresthesia; J. Abdominal Symptoms; K. Nausea and Vomiting; L. Diarrhoea; M. Loss of Consciousness; N. Dizziness; O. Memory Loss; P. Post-traumatic Stress; Q. Lack of Concentration; R. Anxiety; S. Insomnia; T. Nocturia; U. Body Pain; V. Oedema; W. Skin Problem. X. Tinnitus. SR – self-reported symptoms group; SRPF – self-reported symptoms and decreased pulmonary function group.

Supplementary Table 1. Parameters evaluated in the follow-up.

Variable	Control	SR	SRPF	q-value	Adjusted p-value	All p-values
CRP (mg/dL)	3.96 [1.40; 6.98]	5.77 [2.82; 10.45]*	6.08 [3.70; 10.33]*	0.0100; 0.0074	0.0692; 0.0422	<0.01; <0.01; 0.55
D-dimer (mg/L)	410.02 [257.51; 580.05]	418.01 [228.75; 733.17]	463.00 [314.00; 720.00]	n.s.	n.s.	n.s.
Ferritin (μg/L)	182.66 [106.88; 357.69]	131.99 [51.71; 279.07]	115.61 [62.89; 262.45]	n.s.	n.s.	n.s.
Glycated haemoglobin (mmol/mol)	5.57 [5.20; 5.80]	5.77 [5.19; 6.28]	6.24 [5.65; 8.12]*,†	0.0003; 0.0074	0.0003; 0.0338	0.12; <0.01; <0.01
Haemoglobin (g/dL)	14.33 [12.89; 15.42]	13.27 [12.08; 14.41]*	13.02 [11.93; 14.27]*	0.0100; 0.0070	0.0692; 0.0253	<0.01; <0.01; 0.43
Leucocytes	6.46 [5.26; 7.73]	7.08 [6.01; 8.52]*	8.42 [6.20; 9.51]*	0.0485; 0.0013	0.3877; 0.0035	0.02; <0.01; 0.05
MCV (fL)	86.77 [83.33; 88.69]	85.36 [81.11; 88.73]	86.22 [83.02; 89.47]	n.s.	n.s.	n.s.
Platelets (×10^3/μL)	242.35 [197.78; 286.95]	259.10 [213.04; 303.55]	264.00 [219.35; 316.08]	n.s.	n.s.	n.s.
BDNF (pg/mL)	75.42 [51.14; 130.42]	67.28 [48.96; 100.08]	82.66 [58.48; 111.21]	n.s.	n.s.	n.s.
CCL11	15.07 [8.26; 30.30]	9.46 [3.46; 17.54]*	17.43 [11.64; 35.28]†	0.0117; 0.0009	0.0816; 0.0017	<0.01; 0.18; <0.01
EGF	56.50 [32.80; 75.16]	46.37 [22.91; 87.78]	52.39 [25.79; 101.69]	n.s.	n.s.	n.s.
G-CSF	6.55 [2.54; 13.69]	3.29 [2.56; 8.53]	2.84 [2.36; 7.24]*	0.0141	0.1033	0.18; <0.01; 0.09
IFN-α2	6.45 [2.01; 10.82]	2.85 [2.02; 9.41]	2.05 [1.52; 8.91]*	0.0074	0.0422	0.19; <0.01; 0.04
IFN-γ	3.87 [2.51; 7.41]	2.74 [2.60; 7.11]	3.34 [2.57; 5.70]	n.s.	n.s.	n.s.
IL-10	1.99 [1.83; 2.27]	1.98 [1.80; 2.10]	1.87 [1.76; 2.13]	n.s.	n.s.	n.s.
IL-17	1.88 [1.51; 1.97]	1.87 [1.54; 1.96]	1.58 [1.25; 1.95]	n.s.	n.s.	n.s.
IL-4	8.05 [2.95; 74.14]	6.29 [2.89; 46.36]	3.83 [2.38; 16.72]*, †	0.0185; 0.0303	0.1405; 0.2402	0.70; <0.01; 0.01
IL-7	6.77 [4.87; 10.89]	5.96 [3.31; 9.15]	5.59 [3.34; 8.12]	n.s.	n.s.	n.s.
IL-8	20.40 [8.74; 41.40]	21.70 [9.76; 52.00]	24.22 [8.38; 57.03]	n.s.	n.s.	n.s.
IP-10	153.24 [123.24; 203.10]	152.14 [117.02; 245.58]	178.20 [140.44; 228.39]	n.s.	n.s.	n.s.

MCP-1	368.76 [251.10; 534.95]	328.24 [196.12; 426.74]*	363.00 [272.61; 509.90]†	0.0518; 0.0518	0.4254; 0.4254	0.03; 0.82; 0.03
MIP-1α	4.28 [2.26; 6.97]	4.31 [2.41; 7.41]	3.82 [2.24; 10.71]	n.s.	n.s.	n.s.
MIP-1β	11.08 [6.65; 16.69]	10.68 [6.73; 15.60]	14.55 [8.23; 21.96]†	0.0290	0.2262	0.76; 0.03; 0.01
TNF-α	1.58 [1.51; 2.35]	1.63 [1.53; 1.85]	1.81 [1.58; 2.72]	n.s.	n.s.	n.s.
TNF-β	1.73 [1.55; 5.79]	1.69 [1.61; 6.92]	1.68 [1.39; 5.52]	n.s.	n.s.	n.s.
VEGF	28.07 [15.82; 56.47]	26.96 [14.69; 57.77]	26.75 [11.98; 55.92]	n.s.	n.s.	n.s.

Comparisons were performed using the Kruskal-Wallis test followed by Dunn's post hoc test after adjustment by days to follow-up assessment. Statistical significance was considered when q<0.052 in the Dunn's post-hoc test. n.s. non-significant in Kruskall-Wallis test, post-hoc test not performed. BDNF brain-derived neurotrophic factor; BMI body mass index, CCL11 C-C motif chemokine ligand 11 (eotaxin-1); CRP C-reactive protein; EGF endothelial growth factor; G-CSF granulocyte colony-stimulating factor; IFN interferon; IL interleukin MCP1 monocyte chemoattractant protein 1; MCV mean corpuscular volume; MIP macrophage inflammatory protein; TNF tumor necrosis factor; VEGF vascular endothelial growth factor.

Supplementary Table 2. Sex stratification after interval correction for the parameters included in this study.

Variable	Sex	Control	SR	SRPF	q-value	Adjusted p-value	All p-values
Haemoglobin (g/dL)	Women	12.72 [12.12; 13.66]	13.09 [11.98; 14.09]	12.40 [11.54; 13.65]	n.s.	n.s.	n.s.
Haemoglobin (g/dL)	Men	15.00 [14.11; 15.82]	14.29 [12.74; 15.66]	13.80 [12.75; 14.62]*	0.0089	0.0526	0.08; <0.01; 0.23
MCV (fL)	Women	87.21 [82.79; 88.71]	84.71 [80.96; 87.81]	84.10 [82.57; 89.02]	n.s.	n.s.	n.s.
MCV (fL)	Men	86.56 [83.45; 88.58]	86.49 [84.09; 89.52]	87.57 [85.58; 89.91]	n.s.	n.s.	n.s.
Leucocytes	Women	6.30 [4.96; 7.73]	7.32 [6.15; 8.77]*	8.87 [6.31; 10.82]*	0.0549; 0.0018	0.3837; 0.0063	0.02; <0.01; 0.05
Leucocytes	Men	6.56 [5.43; 7.72]	6.87 [5.79; 8.35]	8.03 [6.01; 8.83]	n.s.	n.s.	n.s.
Platelets ($\times10^3/\mu L$)	Women	287.73 [227.40; 305.78]	270.73 [243.53; 317.41]	310.19 [255.81; 346.52]	n.s.	n.s.	n.s.
Platelets ($\times10^3/\mu L$)	Men	232.36 [190.20; 259.19]	219.05 [201.06; 279.66]	224.03 [188.81; 263.03]	n.s.	n.s.	n.s.
CRP (mg/dL)	Women	6.29 [2.96; 8.84]	5.88 [3.58; 10.05]	7.54 [4.88; 13.43]	n.s.	n.s.	n.s.
CRP (mg/dL)	Men	2.81 [1.09; 5.69]	5.42 [1.76; 10.45]	5.19 [3.49; 8.18]	n.s.	n.s.	n.s.
D-dimer (mg/L)	Women	410.02 [273.99; 580.05]	429.50 [237.63; 733.17]	432.97 [334.74; 732.24]	n.s.	n.s.	n.s.
D-dimer (mg/L)	Men	412.51 [233.62; 580.67]	303.77 [187.24; 679.27]	507.53 [310.26; 691.04]	n.s.	n.s.	n.s.
Glycated haemoglobin (mmol/mol)[3]	Women	5.48 [5.25; 5.91]	5.84 [5.38; 6.20]	6.12 [5.50; 8.33]	n.s.	n.s.	n.s.
Glycated haemoglobin (mmol/mol)[3]	Men	5.59 [5.32; 5.92]	5.77 [5.19; 6.94]	6.54 [5.47; 7.58]	n.s.	n.s.	n.s.
Ferritin (µg/L)	Women	106.88 [53.48; 182.66]	104.16 [46.82; 183.95]	102.88 [51.71; 182.54]	n.s.	n.s.	n.s.
Ferritin (µg/L)	Men	281.66 [163.54; 443.32]	283.88 [116.22; 521.32]	261.23 [97.37; 360.76]	n.s.	n.s.	n.s.
BDNF	Women	90 [67; 133]	67 [49; 100]*	87 [61; 114]	0.0549	0.3837	0.02; 0.80; 0.04
BDNF	Men	63 [45; 120]	66 [48; 91]	80 [55; 104]	n.s.	n.s.	n.s.
EGF	Women	67.41 [54.12; 74.34]	45.74 [22.91; 85.70]	51.30 [30.29; 101.53]	n.s.	n.s.	n.s.
EGF	Men	42.73 [29.03; 75.92]	49.74 [23.42; 90.71]	53.47 [13.94; 99.06]	n.s.	n.s.	n.s.
CCL11	Women	13.94 [5.80; 29.74]	6.78 [3.41; 14.24]*	16.14 [11.38; 29.03]†	0.0549; 0.0018	0.3837; 0.0060	0.02; 0.26; <0.01
CCL11	Men	15.77 [8.69; 32.42]	15.29 [6.17; 26.25]	24.74 [12.07; 38.10]	n.s.	n.s.	n.s.

G-CSF	Women	5.92 [2.46; 12.29]	3.35 [2.64; 8.27]	2.82 [2.39; 6.69]	n.s.	n.s.	n.s.
G-CSF	Men	7.18 [2.55; 15.57]	3.21 [2.55; 14.35]	3.04 [2.30; 7.31]	n.s.	n.s.	n.s.
IFN-α2	Women	2.62 [1.94; 8.75]	2.95 [2.06; 7.71]	1.99 [1.44; 6.66]	n.s.	n.s.	n.s.
IFN-α2	Men	7.99 [2.20; 11.52]	2.59 [1.93; 10.52]	2.08 [1.86; 9.97]	n.s.	n.s.	n.s.
IFN-γ	Women	2.68 [2.50; 6.35]	2.68 [2.60; 6.14]	2.64 [2.56; 5.66]	n.s.	n.s.	n.s.
IFN-γ	Men	3.90 [2.58; 7.47]	4.16 [2.61; 9.37]	3.97 [2.59; 6.27]	n.s.	n.s.	n.s.
IL-4	Women	6.18 [3.12; 103.23]	7.81 [3.24; 46.36]	4.07 [2.29; 20.62]	n.s.	n.s.	n.s.
IL-4	Men	8.17 [2.90; 40.62]	4.87 [2.75; 37.13]	3.67 [2.51; 6.64]	n.s.	n.s.	n.s.
IL-7	Women	6.83 [5.17; 12.28]	5.73 [3.33; 7.96]	5.57 [3.34; 9.60]	n.s.	n.s.	n.s.
IL-7	Men	6.60 [4.59; 9.81]	6.67 [2.61; 11.44]	5.59 [3.30; 7.49]	n.s.	n.s.	n.s.
IL-8	Women	18.89 [9.65; 31.33]	24.39 [12.48; 48.99]	21.78 [8.77; 52.34]	n.s.	n.s.	n.s.
IL-8	Men	20.44 [8.66; 52.90]	14.17 [5.90; 56.93]	32.01 [10.21; 58.50]	n.s.	n.s.	n.s.
IL-10	Women	1.96 [1.73; 2.23]	2.00 [1.78; 2.11]	1.87 [1.76; 2.09]	n.s.	n.s.	n.s.
IL-10	Men	2.01 [1.85; 2.43]	1.96 [1.81; 2.06]	1.83 [1.76; 2.21]	n.s.	n.s.	n.s.
IL-17	Women	1.89 [1.51; 1.97]	1.89 [1.77; 1.97]	1.57 [1.24; 1.94]	n.s.	n.s.	n.s.
IL-17	Men	1.87 [1.51; 1.99]	1.56 [1.22; 1.91]	1.59 [1.34; 1.93]	n.s.	n.s.	n.s.
IP-10	Women	156.32 [126.62; 249.15]	150.20 [118.11; 245.58]	175.57 [140.83; 238.93]	n.s.	n.s.	n.s.
IP-10	Men	148.22 [114.16; 194.88]	179.38 [116.44; 251.87]	180.83 [141.05; 218.66]	n.s.	n.s.	n.s.
MCP-1	Women	330.63 [189.72; 454.30]	316.61 [193.08; 438.94]	360.50 [267.63; 535.60]	n.s.	n.s.	n.s.
MCP-1	Men	384.63 [253.10; 550.72]	334.33 [215.36; 392.77]	378.27 [284.44; 499.48]	n.s.	n.s.	n.s.
MIP-1α	Women	4.36 [2.11; 6.97]	4.38 [2.48; 8.51]	4.32 [2.24; 15.05]	n.s.	n.s.	n.s.
MIP-1α	Men	3.81 [2.35; 6.51]	3.00 [2.21; 6.30]	2.89 [2.25; 7.37]	n.s.	n.s.	n.s.
MIP-1β	Women	11.51 [8.77; 14.17]	10.25 [6.36; 14.72]	15.30 [8.85; 26.48]	n.s.	n.s.	n.s.
MIP-1β	Men	9.91 [6.20; 18.22]	12.99 [6.97; 18.91]	13.06 [7.60; 18.55]	n.s.	n.s.	n.s.
TNF-α	Women	1.56 [1.50; 1.93]	1.61 [1.52; 1.77]	1.83 [1.58; 2.51]	n.s.	n.s.	n.s.
TNF-α	Men	1.60 [1.52; 2.44]	1.80 [1.58; 2.35]	1.80 [1.60; 2.83]	n.s.	n.s.	n.s.
TNF-β	Women	1.76 [1.54; 7.20]	1.68 [1.62; 6.92]	1.68 [1.39; 13.39]	n.s.	n.s.	n.s.
TNF-β	Men	1.73 [1.57; 5.42]	1.69 [1.40; 8.93]	1.67 [1.41; 2.18]	n.s.	n.s.	n.s.
VEGF	Women	30.44 [15.45; 59.15]	26.00 [14.69; 53.99]	22.53 [9.43; 57.97]	n.s.	n.s.	n.s.
VEGF	Men	26.46 [16.63; 53.07]	27.41 [14.43; 59.11]	33.63 [17.22; 47.06]	n.s.	n.s.	n.s.

Mean peripheral blood pressure, basal	Women	93.29 [89.64; 100.11]	94.93 [88.70; 104.95]	91.64 [83.97; 104.87]	n.s.	n.s.	n.s.
Mean peripheral blood pressure, basal	Men	91.77 [84.07; 99.41]	93.82 [84.02; 104.88]	88.92 [85.53; 94.25]	n.s.	n.s.	n.s.
Heart rate, basal	Women	71.54 [68.08; 83.44]	79.51 [68.08; 84.17]	80.78 [69.32; 94.55]	n.s.	n.s.	n.s.
Heart rate, basal	Men	67.98 [58.34; 78.47]	74.99 [63.99; 83.41]	70.15 [58.63; 80.43]	n.s.	n.s.	n.s.
respiratory rate, basal	Women	19.06 [17.84; 20.07]	19.97 [18.03; 21.52]	19.96 [18.92; 21.28]	n.s.	n.s.	n.s.
Respiratory rate, basal	Men	19.10 [16.32; 20.09]	18.69 [17.82; 19.99]	19.94 [17.86; 20.54]	n.s.	n.s.	n.s.
Peripheral oxygen saturation, basal	Women	97.07 [94.12; 97.88]	97.34 [96.68; 98.04]	96.89 [95.04; 97.19]†	0.0089	0.0487	0.10; 0.25; <0.01
Peripheral oxygen saturation, basal	Men	97.29 [95.71; 98.02]	96.62 [94.72; 97.09]	97.01 [95.75; 97.70]	n.s.	n.s.	n.s.
Test repetitions	Women	18.91 [13.87; 23.33]	14.45 [12.01; 18.77]	13.60 [10.33; 16.27]	n.s.	n.s.	n.s.
Test repetitions	Men	21.39 [18.51; 25.77]	19.03 [14.15; 24.44]	15.63 [12.16; 18.47]*,†	0.0018; 0.0632	0.0018; 0.4568	0.14; <0.01; 0.03
Borg Dyspnea Scale, pre-test value	Women	0.28 [0.08; 1.95]	1.22 [0.07; 3.02]	1.21 [0.14; 3.03]	n.s.	n.s.	n.s.
Borg Dyspnea Scale, pre-test value	Men	0.06 [-0.03; 0.30]	0.10 [-0.01; 0.96]	1.98 [0.64; 2.92]*,†	0.0018; 0.0633	0.0062; 0.4592	0.18; <0.01; 0.03
Borg Dyspnea Scale, post-test difference	Women	1.56 [0.48; 2.17]	1.95 [0.49; 3.00]	1.56 [0.98; 2.33]	n.s.	n.s.	n.s.
Borg Dyspnea Scale, post-test difference	Men	1.99 [0.47; 2.97]	3.01 [1.00; 3.31]	1.97 [1.08; 2.10]	n.s.	n.s.	n.s.
Mean peripheral blood pressure, post-test difference	Women	10.66 [1.97; 13.32]	2.67 [0.64; 5.96]	0.46 [-4.43; 2.08]	n.s.	n.s.	n.s.
Mean peripheral blood pressure, Post-test difference	Men	-2.34 [-3.32; 1.36]	0.08 [-0.24; 5.71]	4.32 [1.51; 7.14]	n.s.	n.s.	n.s.
heart rate, post-test difference	Women	18.76 [5.96; 32.74]	15.51 [5.56; 25.25]	14.76 [1.67; 19.38]	n.s.	n.s.	n.s.

Heart rate, post-test difference	Men	22.08 [6.24; 30.28]	18.27 [9.01; 26.24]	9.18 [-12.62; 25.90]	n.s.	n.s.	n.s.
Peripheral oxygen saturation, post-test difference	Women	0.72 [0.01; 1.22]	-0.00 [-0.92; 0.83]	-0.05 [-0.99; 1.24]	n.s.	n.s.	n.s.
Peripheral oxygen saturation, post-test difference	Men	0.42 [-0.11; 2.03]	0.01 [-1.01; 1.99]	0.07 [-0.89; 1.04]	n.s.	n.s.	n.s.
Up-and-go test	Women	12.96 [10.69; 14.30]	13.00 [11.24; 15.89]	13.04 [11.95; 16.29]	n.s.	n.s.	n.s.
Up-and-go test	Men	10.62 [9.53; 12.24]	12.40 [11.07; 14.02]*	13.31 [12.30; 15.97]*	0.0591; 0.0027	0.4205; 0.0133	0.03; <0.01; 0.24
Handgrip, left/right hands max	Women	16.54 [8.50; 22.85]	10.09 [4.89; 18.37]	18.13 [15.77; 20.35]†	0.0019	0.0082	0.04; 0.23; <0.01
Handgrip, left/right hands max	Men	32.43 [27.22; 43.47]	34.66 [24.69; 37.98]	27.83 [24.57; 31.05]	n.s.	n.s.	n.s.

Comparisons were performed using the Kruskal-Wallis test followed by Dunn's post hoc test after adjustment by days to follow-up assessment. Statistical significance was considered when q<0.076 in the Dunn's post-hoc test. [a] Adjusted for age. n.s. non-significant in Kruskall-Wallis test, post-hoc test not performed. BDNF brain-derived neurotrophic factor; BMI body mass index, CCL11 C-C motif chemokine ligand 11 (eotaxin-1); CRP C-reactive protein; EGF endothelial growth factor; G-CSF granulocyte colony-stimulating factor; IFN interferon; IL interleukin MCP1 monocyte chemoattractant protein 1; MCV mean corpuscular volume; MIP macrophage inflammatory protein; TNF tumor necrosis factor; VEGF vascular endothelial growth factor.

Supplementary Table 3. Multiple linear regression to test the ability of serum cytokines in predicting the handgrip strength in women. The confounding factors age, sex, and follow-up interval were also included in the model. R^2 = 0.24, F(2.19) = 3.73, p=0.015.

Cytokine	β	*p*-value
BDNF	6,69	0,052
CCL11	4,10	0,060
EGF	1,41	0,448
IFN-α2	-0,59	0,802
IL-4	-1,14	0,293
IL-8	-3,14	0,104
IL-10	10,41	0,100
IL-17	-4,67	0,453
IP-10	-0,28	0,949
MCP-1	1,00	0,750
MIP-1α	-0,49	0,735
MIP-1β	2,56	0,355
TNF-α	11,87	0,039

BDNF – ng/dL, other cytokines – pg/dL.

3 References

1. Johnson WE, Li C, Rabinovic A. Adjusting batch effects in microarray expression data using empirical Bayes methods. Biostatistics [Internet]. 2007 Jan 1 [cited 2023 Mar 27];8(1):118–27. Available from: https://academic.oup.com/biostatistics/article/8/1/118/252073
